# Anip973细胞上清液增加脐静脉内皮细胞的生物活性

**DOI:** 10.3779/j.issn.1009-3419.2015.11.02

**Published:** 2015-11-20

**Authors:** 翠翠 张, 月雅 李, 晶 王, 凯 李

**Affiliations:** 300060 天津，天津医科大学肿瘤医院肺部肿瘤内科，国家肿瘤临床医学研究中心，天津市“肿瘤防治”重点实验室，天津市肺癌诊治中心 Department of Thoracic Oncology, Tianjin Medical University Cancer Institute and Hospital, National Clinical Research Center for Cancer, Tianjin Key Laboratory of Cancer Prevention and Therapy, Tianjin Lung Cancer Diagnosis and Treatment Centre, Tianjin 300060, China

**Keywords:** Anip973, HUVEC, 细胞增殖, 迁移, 成管, CD105, CD31, Annexin Ⅴ, Anip973, HUVEC, Proliferation, Migration, Tube formation, CD105, CD31, Annexin Ⅴ

## Abstract

**背景与目的:**

肿瘤微血管内皮细胞与正常组织来源的微血管内皮细胞相比，具有对生长因子反应性高、粘附因子表达高等特点，造成肿瘤血管通透性高且新生旺盛，导致肿瘤的快速生长和转移。因此了解肿瘤微环境中内皮细胞发生、形态及功能上的异质性特点，对进行合理的、个性化的、以血管内皮细胞为靶点的抗血管新生治疗有一定的指导作用。本实验旨在研究高转移肺腺癌Anip973细胞培养上清液对人脐静脉内皮细胞(human umbilical vein endothelial cell, HUVEC)表面标记及其生物学行为的影响。

**方法:**

以含有不同浓度Anip973细胞上清液的培养基(RPMI-1640+10%胎牛血清)培养的HUVEC为实验组、单纯培养基培养的HUVEC为对照组，以CCK-8检测各组细胞增殖率、基质胶成管实验检测成管能力、Transwell检测迁移能力、流式细胞术检测细胞表面标记CD105、CD31以及凋亡标记Annexin Ⅴ的表达，并分析细胞表面标记的表达与生物学行为之间的关系。

**结果:**

与对照组相比，经Anip973细胞上清液培养的HUVEC增殖更多、且在浓度为250 μL/mL作用24 h最明显(*P*=0.002)；成管及迁移能力亦均较对照组增强，分别在浓度为125 μL/mL(*P*＜0.001)、250 μL/mL(*P*=0.002)时达最强；细胞表面标记CD105表达阳性率升高、浓度为250 μL/mL(*P*=0.028)最明显；CD31表达阳性率呈浓度依赖性升高；凋亡细胞比例降低。相关性分析显示：CD105的表达阳性率与细胞增殖及迁移能力呈正相关关系、CD31的表达与生物学行为并无明显相关性。

**结论:**

Anip973细胞上清液能促进HUVEC细胞增殖、迁移、血管形成，CD105也随之变化、并可在一定程度上反应其生物学行为。

## Abstract (en)

**Background and objective:**

Unlike normal tissue-derived microvascular endothelial cells, tumor microvessel endothelial cells are highly reactive to growth factors and exhibit more adhesion molecules. Thus, vascular tumors are highly permeable and grow vigorously; this occurrence results in rapid growth and metastasis cancer cells. Therefore, understanding the characteristics of endothelial cells in the tumor microenvironment guides anti-angiogenic therapy. To this end, we explore the effect of the supernatant obtained from cultured Anip973 cells (high-metastatic human lung adenocarcinoma cells) on the biological behavior and on the cell surface markers of the human umbilical vein endothelial cell (HUVEC).

**Methods:**

The HUVEC that was cultured in a medium (RPMI-1640 + 10% fetal bovine serum) containing various concentrations of Anip973 supernatants was categorized into experimental groups. The HUVEC cultured in a medium without Anip973 supernatants served as the control group. Proliferation was determined with CCK-8; blood vessel formation was investigated with three-dimensional culture techniques in vitro; and HUVEC migration was observed via transwell assay. At the same time, the expressions of CD105, CD31, and the apoptotic marker of Annexin Ⅴ were detected through flow cytometry for analyzing the relationship between the expression of cell surface markers and biological behavior.

**Results:**

Following incubation with the supernatant obtained from cultured Anip973 cells, HUVEC proliferated more than the control group did, and the proliferation rate was maximized when incubated in a supernatant concentration of 250 μL/mL for 24 h (*P*=0.002). In addition, the experimental groups exhibited varying degrees of migration and forms of vascular lumen sample structure, especially at supernatant concentrations of 125 μL/mL (*P* < 0.001) and 250 μL/mL (*P*=0.002), respectively. CD105 expression was optimized at 250 μL/mL (*P*=0.028), and CD31 expression also increased with an increase in concentration. However, the percentage of apoptotic cells decreased. Correlation analysis results showed that cell proliferation, migration, and CD105 expression were significantly and positively correlated with one another. By contrast, no significant correlation was detected between CD31 expression and biological behavior.

**Conclusion:**

Anip973 supernatants can promote HUVEC proliferation and migration, as well as angiogenesis. In addition, cell surface markers can change concurrently and relatively. To a certain extent, changes in CD105 expression can be attributed to shifts in its biological behavior.

肿瘤微环境包括肿瘤细胞、基质细胞(内皮细胞、成纤维细胞、平滑肌细胞、周细胞、炎性细胞等)、细胞外基质及其分泌或释放的各种细胞因子，是肿瘤形成、生存和增殖之场所。肿瘤生长需要新生血管生成以持续供氧和营养，其分泌的基质金属蛋白酶(matrix metalloproteinase, MMP)、血管内皮生长因子(vascular endothelial growth factor, VEGF)、碱性成纤维细胞生长因子(basic fibroblast growth factor, bFGF)等促血管生成因子可诱导微血管生成，而生成过程不仅与血管内皮细胞的增殖及细胞外基质降解有关，还依赖血管内皮细胞的迁移并形成管样结构^[1]^。肿瘤细胞培养上清液中含有大量其产生的因子、与人体中的微环境有相似性，故可通过研究肿瘤细胞上清液对内皮细胞生物学效应的影响推测体内微环境中内皮细胞的生物学行为。本实验观察了高转移人肺腺癌细胞Anip973细胞上清液对人脐静脉内皮细胞(human umbilical vein endothelial cell, HUVEC)生物学行为及细胞表面标记表达的影响。

## 1 材料与方法

### 1.1 细胞来源

高转移肺腺癌细胞系Anip973购自上海赛默科技发展有限公司；HUVEC购自北京金紫晶生物医药技术有限公司。

### 1.2 试剂及仪器

RPMI-1640培养液、胎牛血清(fetal bovine serum, FBS)、青霉素/链霉素双抗(美国GIBCO公司)；CCK-8试剂盒(日本同仁化学研究所)；Transwell chamber(美国Corning Costar公司)；anti-CD105-FITC、荧光素FITC标记的膜联蛋白Ⅴ(Annexin Ⅴ-FITC美国BioLegend公司)；Matrigel基质胶、anti-CD31-PE(美国BD公司)。CO_2_细胞培养箱(日本SANYO公司)；倒置荧光相差显微镜(日本Nikon公司)；Nanodrop3300紫外分光光度计(美国Thermo公司)；流式细胞仪(EPICs-XL)(美国Beckman Coulter公司)。

### 1.3 方法

#### 1.3.1 细胞培养

将Anip973、HUVEC常规培养于含10%胎牛血清的RPMI-1640培养液中，置于37 ℃含5%CO_2_培养箱中培养。取对数生长期的Anip973细胞，调整细胞浓度为2×10^5^个/mL于37 ℃含5%CO_2_培养箱中培养24 h，收集上清液，用含有10%胎牛血清的RPMI-1640培养液配置成含Anip973上清浓度为62.5 µL/mL、125 µL/mL、250 µL/mL、500 µL/mL的培养液备用。

#### 1.3.2 CCK-8法检测Anip973上清液对HUVEC增殖的影响

取对数生长期细胞配制成细胞数量约为1×10^5^个/mL的细胞悬液置于37 ℃含5%CO_2_培养箱中培养，待细胞贴壁后吸出上清，实验组加入上述含不同浓度Anip973上清液的培养液，对照组则加入单纯培养液，置入37 ℃细胞培养箱中孵育24 h、48 h、72 h，孵育结束后取出加入CCK-8溶液、混合均匀，于37 ℃细胞培养箱中孵育1 h。置入分光光度计中测定各孔450 nm处的光密度(optical density, OD)值，计算每组OD平均值。

#### 1.3.3 管状结构形成实验

将Matrigel基质胶、无菌96孔平板、枪头等置于4 ℃冰箱内预冷，取Matrigel胶50 µL/孔涂布于96孔平板，冰上放置20 min使基质胶分布均匀，然后置于37 ℃孵箱中30 min使基质胶凝固；将对数生长期HUVEC消化成单细胞悬液，计数为5×10^5^个/mL、100µL/孔接种于Matrigel表面，对照组加入新鲜培养基，实验组则加入含不同浓度Anip973上清液的培养液，各孔终体积为200 µL。置于37 ℃含5%CO_2_培养箱中培养，分别于4 h、8 h、12 h后倒置相差显微镜下观察并摄片细胞间连接形成的中空管样结构，计数。

#### 1.3.4 Transwell迁移实验

将Transwell小室放入培养板中，下室中加入600 µL含20%FBS的RPMI-1640作为趋化剂，在上室内加入100 µL HUVEC细胞悬液。培养箱中培养6 h，取出小室，用预冷的无水甲醇固定15 min，10%Giemsa染色15 min，在显微镜下随即选取5个视野计数迁移至滤膜下表面的细胞数。

#### 1.3.5 流式细胞术

按常规方法消化、离心、收集细胞于流式管中，实验管分别加入FITC标记的CD105、PE标记的CD31，对照管加入FITC及PE同型对照；Annexin Ⅴ为FITC标记，用同样方法标记，室温避光孵育30 min，清洗细胞1次，流式细胞仪检测各指标的表达率。

#### 1.3.6 统计学方法

采用SPSS 17.0统计学软件分析。正态分布的计量资料采用单因素方差分析，非正态分布的计量资料采用秩和检验。相关性分析：双变量正态分布资料选用*Pearson*相关检验，非双变量正态分布资料，选用*Spearman*相关检验，以*P*＜0.05为差异有统计学意义。

## 2 结果

### 2.1 Anip973细胞上清液对HUVEC增殖的影响

不同浓度的细胞上清液在作用24 h时对HUVEC增殖均有促进作用、以250 µL/mL时最明显(*P*=0.002)(图 1)。而在作用48 h及72 h时HUVEC增殖率并未再继续增加，在500 µL/mL作用72 h甚至出现了抑制作用。因此，后续的Transwell迁移实验及流式细胞术选取Anip973培养上清液作用24 h的HUVEC进行。

**1:**
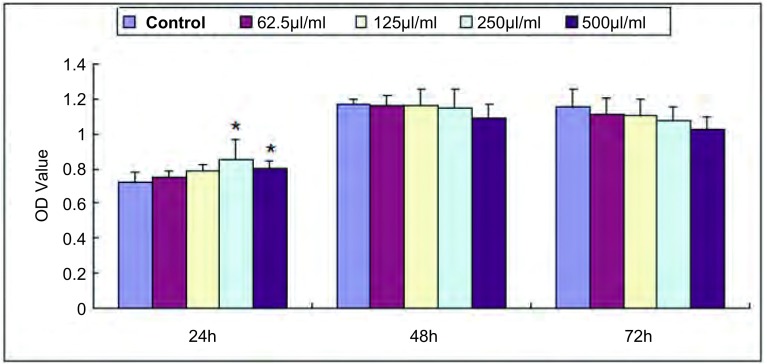
Anip973上清液对HUVEC增殖的影响(*x*±s)。^*^：与对照组比较差异有统计学意义，*P*＜0.05。 The effect of supernatant from cultured Anip973 cells on cell proliferation of HUVEC (*x*±s). ^*^: Compared with the control, *P* < 0.05.

### 2.2 管状结构形成实验观察管状结构形成

各组细胞在4 h即可形成中空的管状结构，12 h后逐渐减少、消失，因此取4 h、8 h、12 h作为观察点。上清液干预组中空样管状结构较对照组明显增多，在浓度125 µL/mL时最多(图 2，图 3)。

**2:**
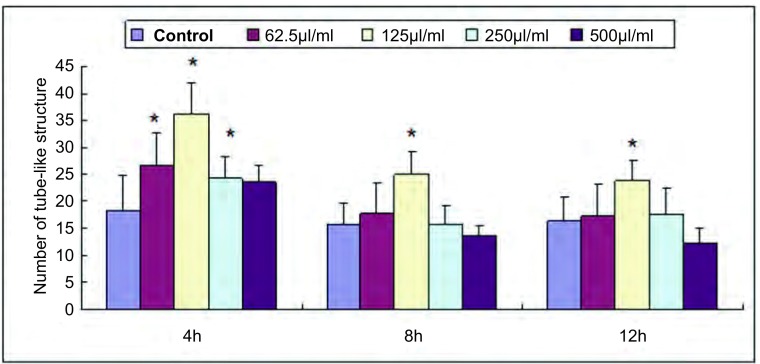
Anip973上清液对HUVEC成管数目的影响(*x*±s)。^*^：与对照组比较差异有统计学意义，*P*＜0.05。 The effect of supernatant from cultured Anip973 cells on angiogenesis of HUVEC (*x*±s). ^*^: Compared with the control, *P* < 0.05.

**3:**
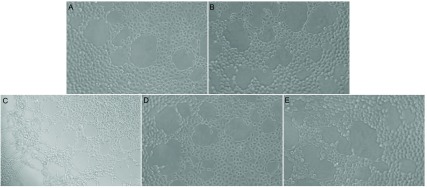
应用HUVEC三维培养观察各组细胞中空样管状结构形成(×200)。A：对照；B：Ainp973上清62.5 µL/mL；C：Anip973上清125 µL/mL；D：Anip973上清250 µL/mL；E：Anip973上清500 µL/mL。 HUVEC tube formation (×200). A: Control; B: Anip973 supernatant 62.5 µL/mL; C: Anip973 supernatant 125 µL/mL; D: Anip973 supernatant 250 µL/mL; E: Anip973 supernatant 500 µL/mL.

### 2.3 Transwell实验检测细胞迁移能力

与对照组相比，经Anip973细胞上清液干预后，各实验组迁移细胞数目均增多，在浓度为250 µL/mL、500 µL/mL时与对照组比较差异有统计学意义(图 4、图 5)。

**4:**
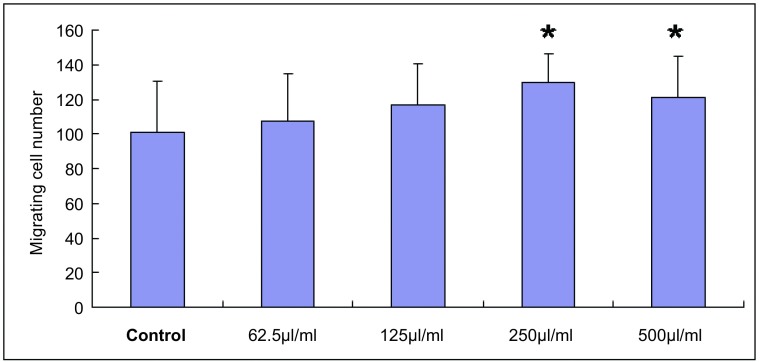
Transwell实验检测HUVEC迁移能力结果。^*^：与对照组比较差异有统计学意义，*P*＜0.05。 Ability of migration was assayed using Transwell. ^*^: compared with the control, *P* < 0.05.

**5:**
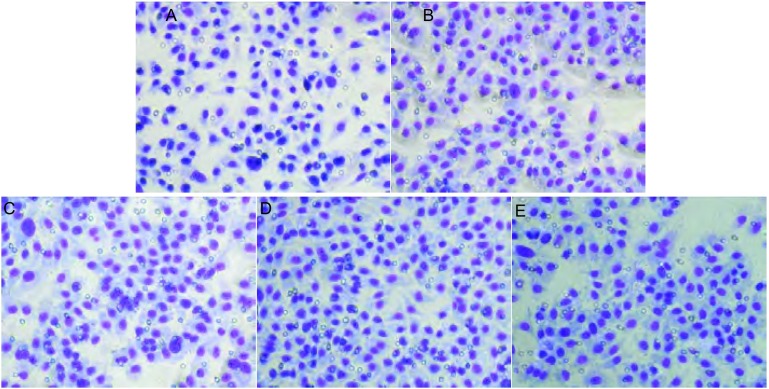
Transwell实验观察HUVEC迁移能力(×400)。A：对照；B Ainp973上清62.5 µL/mL；C：Anip973上清125 µL/mL；D：Anip973上清250 µL/mL；E：Anip973上清500 µL/mL。 Ability of migration was assayed using Transwell (×400). A: Control; B: Anip973 supernatant 62.5 µL/mL; C: Anip973 supernatant 125 µL/mL; D: Anip973 supernatant 250 µL/mL; E: Anip973 supernatant 500 µL/mL.

### 2.4 流式细胞术检测细胞表面标记

HUVEC经Anip973细胞上清液作用24 h后CD105阳性表达率升高、在250 µL/mL时最高；CD31表达阳性率呈浓度依赖性升高；而Annexin Ⅴ下降、在浓度为250 µL/mL时最低(图 6)。

**6:**
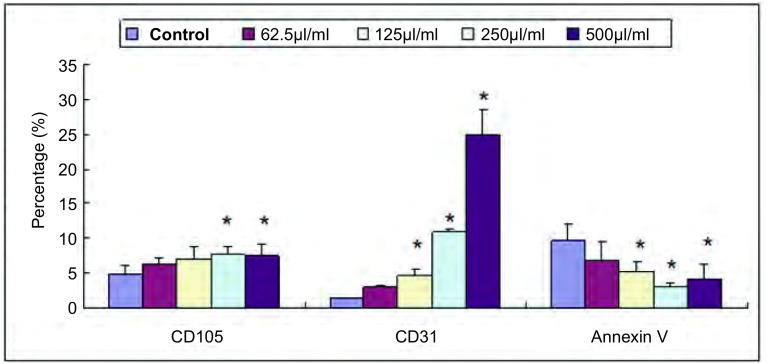
Anip973上清液对HUVEC细胞表面标记表达率的影响(%，*x*±s)。^*^：与对照组比较差异有统计学意义，*P*＜0.05。 Effects of supernatant from cultured Anip973 cells on HUVEC surface markers (%, *x*±s). ^*^: compared with the control, *P* < 0.05.

### 2.5 相关性分析

经相关性分析发现，细胞表面标记CD105表达阳性率与HUVEC增殖能力及迁移能力呈正相关关系(*P*=0.017)，而与成管能力无关；CD31表达与生物学行为无明显相关(表 1)。

**1:** 细胞表面标记与生物学行为的相关性分析 The correlation analysis between cell biological activities and surface maker

Detection index		Proliferation	Migration	Angiogenesis
CD105	*r*	0.942	0.941	0.521
	*P*	0.017	0.017	0.368
CD31	*r*	0.619	0.614	0.036
	*P*	0.266	0.270	0.954

## 3 讨论

肿瘤组织中新生血管提供持续的营养供应并排泄代谢产物，对实体瘤的生长十分重要^[2]^。血管内皮细胞的增殖是新生血管形成的重要环节。McMullen等^[3]^指出，VEGF可通过p38-MAPK途径促进内皮细胞的迁移。另有研究证实^[4, 5]^，在非小细胞肺癌肿瘤血管的生成过程中，VEGF促进内皮细胞增殖、Ang-2影响支持细胞的募集，二者具有协同作用，共同参与调节新生血管的生成。李白翎等^[6]^发现，血管紧张素(Ang)家族与血管内皮细胞的迁移有关，且当有VEGF存在时，Ang-2可以促进内皮细胞的增殖、迁移及血管形成。上述研究提示多种促血管生成因子可协同促进内皮细胞增殖、迁移。本实验利用不同浓度高转移肺腺癌细胞Anip973培养上清液培养HUVEC，观察其对HUVEC行为及表面活性标记的影响，旨在模拟体内环境、阐明肿瘤细胞增强血管内皮细胞活性及促进血管生成的过程。

我们发现Anip973培养上清液对HUVEC具有促分裂增殖作用，与文献^[7]^报道一致。郑苇杭等^[8]^认为肿瘤细胞上清液促进HUVEC增殖的机制是其中的细胞因子激活周期蛋白D激酶、导致Cyclin D和CDK高表达，诱导更多细胞进入S期、增加DNA合成。本实验结果还显示上清液培养的HUVEC凋亡率较对照组降低，与李白翎等^[6]^的实验结果相似。然而究竟是何种细胞因子促进细胞增殖、抑制凋亡，某一因子的最佳作用浓度为何，上清液中各因子浓度与患者血中的浓度是否一致等问题仍待进一步研究。

肿瘤血管生成的主要步骤还包括内皮细胞的迁移及管状血管结构的形成；没有迁移及形成管状结构的能力，增殖活跃的血管内皮细胞只能形成杂乱无章的细胞团、而非能提供氧气及营养的血管。内皮细胞通过伪足形成实现迁移^[9]^，本实验发现，经肿瘤细胞上清液作用后HUVEC迁移及形成管状结构能力增强，提示肿瘤细胞分泌的促血管生成因子可以通过增强内皮细胞的迁移成管能力来促进肿瘤新生血管的形成，但其是否能直接形成血管内皮细胞伪足仍待证实。

因为在活体中检测血管内皮细胞生物学行为相对困难，我们试图寻找与肿瘤血管内皮细胞生物学行为相关的标志，以动态监测血管生成。本实验中选取介导内皮细胞增殖、分裂的指标CD105^[10]^、与内皮细胞之间粘着相关的CD31^[10]^以及凋亡标记Annexin Ⅴ^[11]^。结果显示肿瘤细胞上清液促进CD31、CD105表达、降低Annexin Ⅴ表达，与权琳等^[12]^的非小细胞肺癌患者外周血中循环血管内皮细胞(CD45^-^CD146^+^CD105^+^CD31^+^)含量高于正常对照组的临床研究结论相似。相关性分析发现，细胞表面标记CD105表达率与HUVEC增殖、迁移能力呈正相关；Fonsatti等^[13]^报道CD105可调节信号受体Ⅰ与信号受体Ⅱ与肿瘤转化生长因子-β(transforming growth factor-β, TGF-β)相互反应、使TGF-β能失活而对抗TGF-β对组织生长及肿瘤血管内皮细胞增殖的抑制作用，从而促进了组织的生长与血管内皮细胞的增殖；同时亦能直接参与细胞周期，促进肿瘤血管内皮细胞的增殖并抑制凋亡^[14]^。而CD105与细胞迁移能力的关系却未见报道、有待继续探索。但CD31表达与HUVEC的增殖、迁移及成管能力等生物学行为并无相关性，与Matsumura等^[15]^的报道并不相符，可能系因HUVEC粘附、成管能力受多种细胞因子共同作用的影响，单独检测一种并不能代表整个生物学行为的改变；亦可能缘于导致其生物学行为的改变与CD31表达阳性率改变的最适浓度不同所致。我们将增加Anip973细胞上清液浓度梯度，同时检测钙粘蛋白等粘附相关指标，试图寻找出与内皮细胞粘附成管能力相关的、易于检测的生物学指标。

综上所述，本实验结果提示肿瘤细胞上清液可促进血管内皮细胞分裂增殖、迁移成管，并引起细胞表面标记出现相应变化，提示有望通过细胞表面标记推测细胞生物学行为的变化。
